# Right ventricular mechanical pattern in health and disease: beyond longitudinal shortening

**DOI:** 10.1007/s10741-019-09778-1

**Published:** 2019-03-09

**Authors:** Attila Kovács, Bálint Lakatos, Márton Tokodi, Béla Merkely

**Affiliations:** Semmelweis University Heart and Vascular Center, Városmajor St. 68, Budapest, H-1122 Hungary

**Keywords:** Right ventricle, 3D echocardiography, Speckle tracking, Heart failure, Pulmonary hypertension

## Abstract

Right ventricular (RV) function has proven to be a prognostic factor in heart failure with reduced and preserved ejection fraction and in pulmonary hypertension. RV function is also a cornerstone in the management of novel clinical issues, such as mechanical circulatory support devices or grown-up congenital heart disease patients. Despite the notable amount of circumferentially oriented myofibers in the subepicardial layer of the RV myocardium, the non-longitudinal motion directions are often neglected in the everyday assessment of RV function by echocardiography. However, the complex RV contraction pattern incorporates different motion components along three anatomically relevant axes: longitudinal shortening with traction of the tricuspid annulus towards the apex, radial motion of free wall often referred as the “bellows effect”, and anteroposterior shortening of the chamber by stretching the free wall over the septum. Advanced echocardiographic techniques, such as speckle-tracking and 3D echocardiography allow an in-depth characterization of RV mechanical pattern, providing better understanding of RV systolic and diastolic function. In our current review, we summarize the existing knowledge regarding RV mechanical adaptation to pressure- and/or volume-overloaded states and also other physiologic or pathologic conditions.

## Introduction

Compared to the left ventricle (LV), whose anatomy and function has been the subject of intensive research, right ventricular (RV) morphology and mechanics were traditionally less studied in contemporary science. In the past few decades, novel diagnostic techniques and important epidemiology studies have brought RV function back into the scientific spotlight. RV function has proven to be a prognostic factor in heart failure with reduced and preserved ejection fraction and in pulmonary hypertension. RV function is also a cornerstone in the management of novel clinical issues, such as mechanical circulatory support devices or grown-up congenital heart disease patients. In our current review, we summarize the existing knowledge regarding RV mechanics in different overload conditions and emphasize the role of non-longitudinal contraction of the chamber and its evaluation.

## Myofiber architecture of the RV

**Fig. 1:**
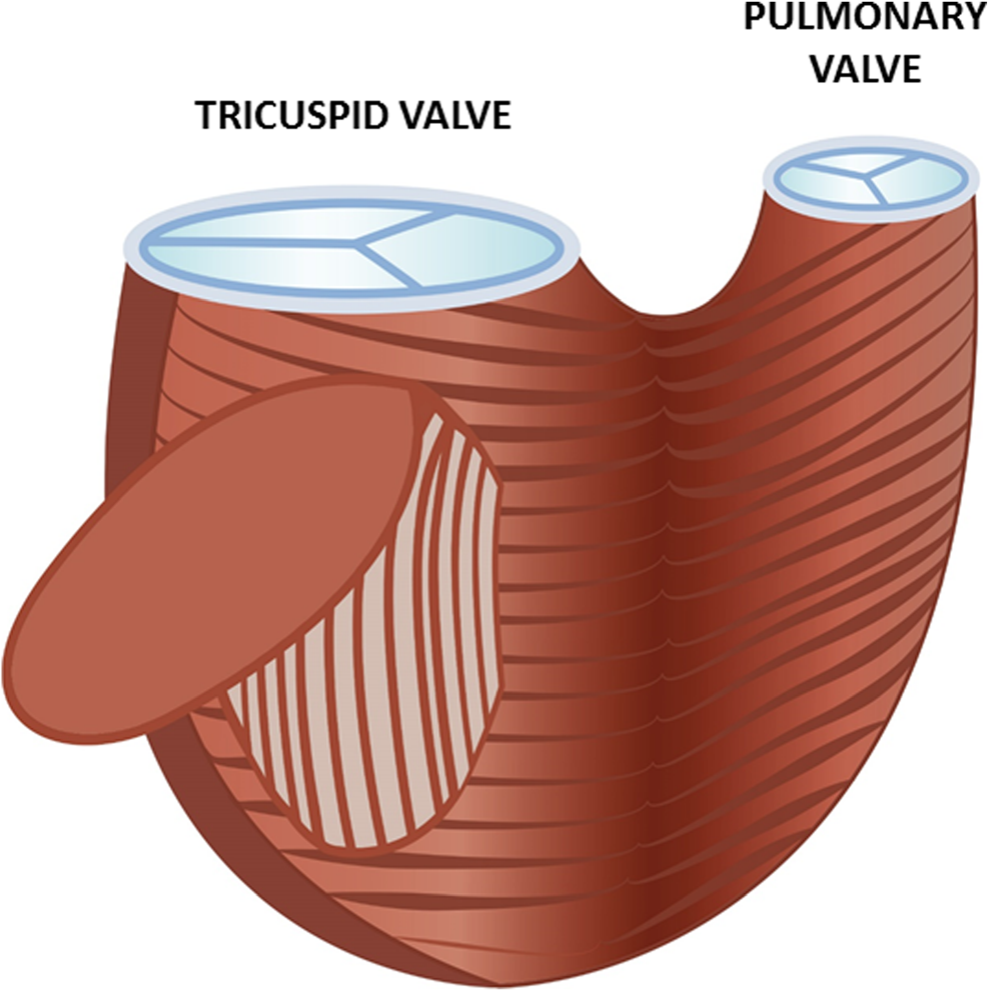
Schematic representation of the right ventricular myofiber orientation. On the epicardial surface, circumferentially oriented myofibers are present; these myofibers encompass the subpulmonary infundibulum and advance more or less parallel with the atrioventricular groove. At the apex, this layer spirals into the deep layer of the RV. This subendocardial fiber orientation is rather longitudinal (as shown inside the cavity)

The myocardium is a complex three-dimensional network of myofibers in a multiple helical arrangement with important functional consequences. A contraction of these bundles deforms the ventricle and generates ejection, whereas the relaxation aids diastole; moreover, knowing the myofiber orientation helps to understand the pattern of RV contraction in the three-dimensional space. On the epicardial surface, circumferentially oriented myofibers are present; these myofibers are components of the myofiber tracts that are shared with the LV, encompass the subpulmonary infundibulum and advance more or less parallel with the atrioventricular groove. At the apex, this layer spirals into the deep (subendocardial) layer of the RV. However, this subendocardial fiber orientation is rather longitudinal (Fig. 1). In physiologic conditions, the mid-layer containing circumferential fibers is absent [1]. However, there are congenital and acquired modifications of this myofiber architecture that result in an altered motion pattern of the RV.

## Functional pattern of RV myocardial mechanics

Mechanistically, the RV shows a distinctive, peristaltic-like contraction pattern: the activation starts from the inlet portion and ends at the infundibulum [2]. During the isovolumic contraction, the subepicardial layer of the inflow tract acts as an early pressure generator and deforms the RV circumferentially. Subendocardial fibers are responsible for the longitudinal shortening mainly during the ejection phase [3]. The function of the interventricular septum also accounts for a significant part of global RV function, mainly through its longitudinal shortening. Notably, an inward motion of the septal myocardium into the RV cavity can also develop. There is no significant contribution of twist to RV pump function [4].

Accordingly, three main mechanisms contribute to RV pump function: (1) shortening of the longitudinal axis with traction of the tricuspid annulus towards the apex; (2) inward (radial) movement of the RV free wall, which is often referred as the “bellows effect”; and (3) bulging of the interventricular septum into the RV during the left ventricular contraction and stretching of the free wall over the septum (causing shortening in the anteroposterior direction).

**Fig. 2:**
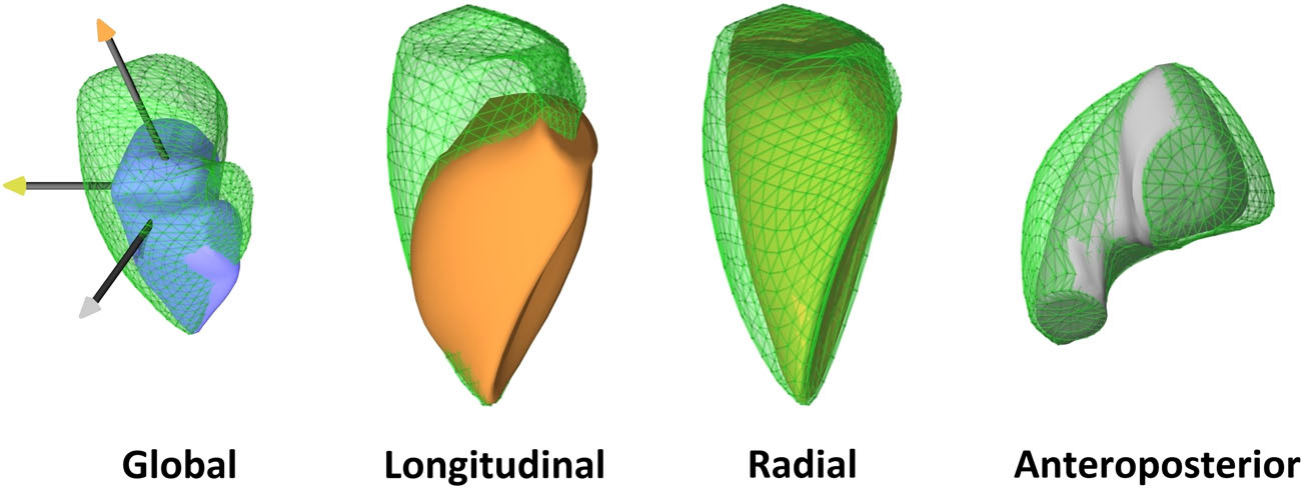
Global function and decomposed motion of the right ventricle in a healthy subject (end-diastolic volume 92 mL, ejection fraction 63%). Shortening along the longitudinal axis is relatively easy to appreciate; however, significant systolic motion along the other two directions can also be observed by decomposing the motion of the right ventricle. By quantifying the relative contribution of these components, they appear to be roughly equal (longitudinal ejection fraction 24%, radial ejection fraction 25%, anteroposterior ejection fraction 23%)

Normative data are still lacking regarding the relative contribution of the longitudinal, radial, and anteroposterior components of RV wall motion to global ejection. Under physiological conditions, the longitudinal shortening was suggested to account for the majority of RV pump function [5]; however, the majority of recent studies suggest a similar importance of longitudinal and radial motions (Fig. 2) [6, 7]. Larger studies are needed to characterize potential age-related and gender-related alterations. Moreover, there are several diseases and clinical scenarios in which the normal ratio between the different mechanisms can change, indicating the (mal)adaptation of the chamber.

## Advanced echocardiographic techniques to characterize RV mechanics

Two-dimensional echocardiography is barely capable of thoroughly characterizing RV morphology and function. Still, RV function assessment is often based exclusively on tricuspid annular plane systolic excursion (TAPSE), which refers only to the longitudinal shortening of the chamber. The estimation of RV volumes based on 2D geometrical assumptions (as regarding the LV) are not used routinely; instead, fractional area change (FAC) is calculated. Although this technique is a 2D approach and partially incorporates radial motion direction, FAC describes RV function in a single cut of a very complex 3D structure. Speckle-tracking echocardiography emerged as a valuable tool for a more quantitative, semi-automated approach. Global longitudinal strain is showing an established diagnostic and prognostic value regarding LV pathologies [8], and promising data also have been gathered for the RV [9]. Although the RV longitudinal strain has an established added prognostic value compared to TAPSE, longitudinal strain still incorporates only one motion direction.

As a recent advancement, 3D echocardiography may overcome these shortcomings. Several software solutions are available to reconstruct the endocardial surface of the RV for a better appreciation of its geometry and to allow the measurement of volumes and subsequent ejection fraction. In addition, novel postprocessing solutions have become available for a deeper analysis of shape and function. Beyond 3D speckle tracking, curvature analysis and the separate quantification of the different motion components (longitudinal, radial, and anteroposterior) are promising in terms of obtaining a deeper understanding of RV physiology and pathophysiology [10, 11].

## Pressure overload of the RV

**Fig. 3:**
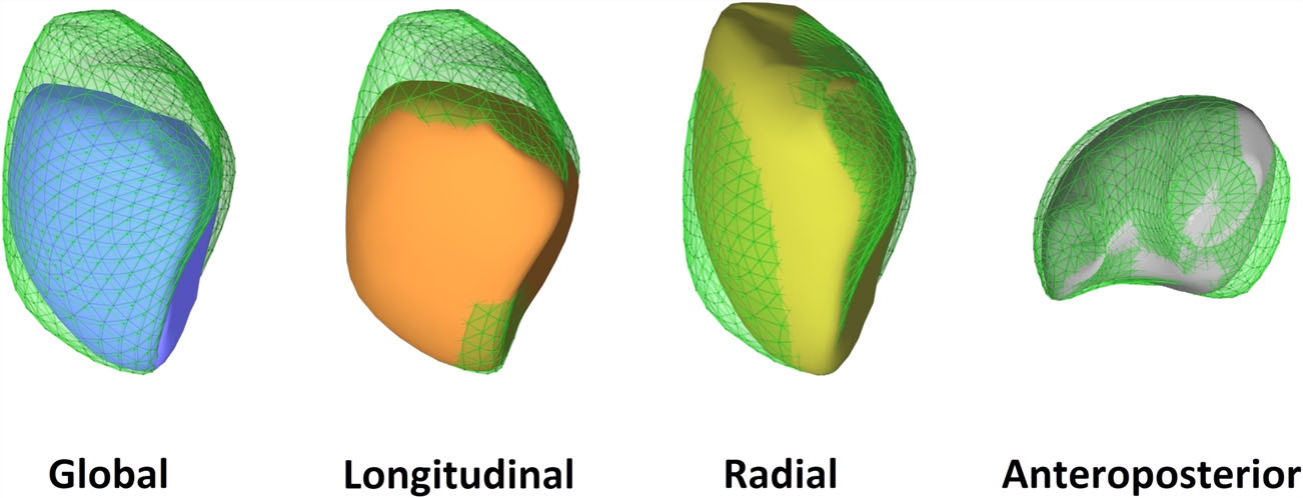
Representative case of a patient with pulmonary arterial hypertension (pulmonary vascular resistance 9.6 Wood units). The right ventricle is severely dilated (end-diastolic volume 234 mL) and global right ventricular function is decreased (ejection fraction 34%). By decomposing the motion of the right ventricle, a relatively preserved longitudinal function can be seen (longitudinal ejection fraction 17%), while radial (radial ejection fraction 8%) and anteroposterior shortening (anteroposterior ejection fraction 6%) is decreased. The basal predominance of right ventricular dysfunction is also notable

In conditions accompanied by pressure overload, the challenge for the RV is to remain coupled to the increased afterload. The major mechanisms include adaptive myocardial hypertrophy, which leads to increased wall thickness and reduced wall stress, and alterations of muscle properties, such as myocardial fiber orientation and also RV shape [10]. The relative dominance of the circumferential fibers within the RV wall can be observed [12]. A greater decline in radial contraction is often reported (Fig. 3), and radial motion seems to be a better predictor of RV pump function and of pulmonary artery pressure than longitudinal shortening [13]. Moreover, recent studies have demonstrated the prognostic value of the “bellows effect” in pulmonary hypertension patients [14].

RV-LV interactions should not be neglected in pulmonary hypertension. There is a parallel interaction via the leftward bowing of the interventricular septum and an in-series interaction as the RV fails to supply an adequate LV preload. Another interesting aspect of ventricular interdependence is functional linkage via common myofibers. In experimental models of pulmonary artery banding, the addition of a mild aortic band increases the contractility of the pressure-stressed RV, and this change is probably attributable to the Anrep effect and the continuity of myofibers [15]. Better coordination of RV and LV contractions and temporal alignment of cardiac cycle events can improve function, as also seen in bundle branch block activation delay.

Although pressure overload induces prominent changes in RV structure and function, RV remodeling seems to be highly reversible, and the recovery of RV function could be observed after appropriate treatment. Patients with pulmonary hypertension showed markedly improved RV function and decreased RV wall thickness after lung transplantation, even with severe preoperative RV dysfunction [16]. The degree of improvement is variable and related to pulmonary artery pressure and the duration of disease prior to transplantation [17]. The regaining of radial contraction may serve as a sensitive marker of disease regression during follow-up in these patients.

## Volume overload of the RV

When RV preload increases, wall stress is maintained by dilation of the chamber, along with preserved end-systolic volumes in the early stages; these observations suggest increased contractile function through the Frank-Starling law, which effectively compensates for altered hemodynamic conditions. Clinical experience includes an accompanying increase in RV longitudinal shortening. The significant elevation of RV preload may be also tolerable with maintained global function in acute and chronic settings, and these observations led to the conclusion that RV volume overload is a benign condition that lacks hemodynamically detrimental consequences. However, there is growing evidence from tricuspid and pulmonary regurgitation patients that strongly emphasizes the negative effects of RV volume overload [18–21].

In the clinical setting, the main etiologic factors of RV volume overload are tricuspid regurgitation (TR), pulmonary valve regurgitation, and left-to-right shunts. The latter two entities are almost exclusively associated with congenital heart diseases; for such diseases, in addition to the presence of RV volume overload, other complex structural and functional aspects must be taken into consideration and will be discussed later. Approximately 80% of TR is functional, and the nature of the primary disease also significantly affects pathophysiology and presentation [20]. The majority of experimental TR models and clinical data are not focused on RV mechanics and/or mechanoenergetics. According to the available experimental data, acute, severe TR results in elevated RV end-diastolic volume, along with depressed RV performance, which also persists in the subacute phase [19]. A better understanding of RV function in TR would be of interest, considering that the independent prognostic significance of TR is well known in a wide spectrum of cardiovascular disorders, and the current management of these patients is still a matter of debate [20].

## Congenital heart diseases, arrhythmogenic cardiomyopathy

Congenital heart diseases (CHD) represent a wide range of cardiac malformations, in which RV characteristics are also the cornerstones of disease presentation and progression. Depending on the nature of the abnormality, the RV may be exposed to severe pressure and/or volume overload. In certain types of defects, the myofiber architecture also differs from the physiological architecture [22], and this effect may become more prominent based on the altered loading conditions of the RV [12]. Because of these complex factors, CHD patients present diverse forms of RV geometry and mechanics.

In isolated atrial septal defect (ASD) patients, increased preload results in the dilation of the RV, along with maintained/increased contractility in compensated state [23]. Global RV function measured by the RV ejection fraction is usually preserved or even supernormal. Speckle-tracking analysis and tissue Doppler imaging reveal higher global longitudinal strain (GLS) and systolic velocity in ASD patients than controls, suggesting that the increased longitudinal shortening, especially in the apical segments, may be attributable to the increased systolic function [24]. When a patient’s shunt volume is high and closure of the defect is not performed, severe volume overload induces pulmonary hypertension with fixed near-systemic resistance of the pulmonary vascular bed (Eisenmenger’s syndrome) [25]. Compared to idiopathic pulmonary arterial hypertension, CHD-associated pulmonary hypertension is associated with more pronounced concentric RV hypertrophy [26]. Beyond morphological differences, RV mechanics are also markedly different in these patients; in contrast to the severely decreased RV radial shortening observed in pulmonary arterial hypertension patients, in Eisenmenger’s syndrome, the transverse (radial) function of the RV is relatively preserved, predominantly in patients with posttricuspid shunts [26]. Moreover, transverse strain of the RV is an independent predictor of mortality in this population [26].

**Fig. 4:**
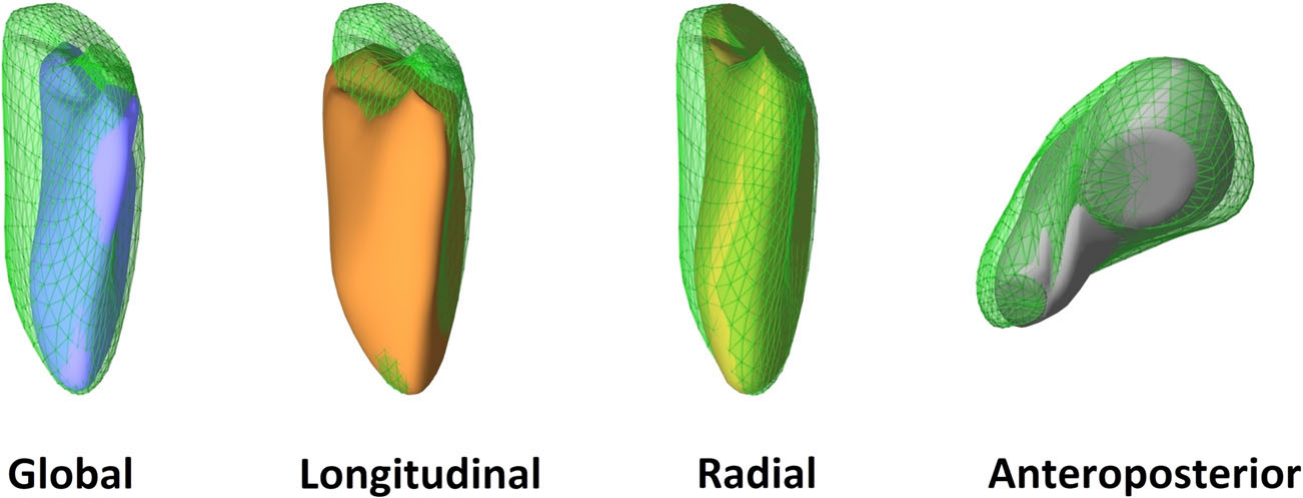
Representative case of a surgically repaired tetralogy of Fallot patient with severe pulmonary regurgitation. The right ventricle is dilated (end-diastolic volume 187 mL) predominantly by elongation of the chamber. Global right ventricular function is in the low-normal range (ejection fraction 48%); however, longitudinal shortening is severely depressed (longitudinal ejection fraction 11%) compared to radial function (radial ejection fraction 27%), while anteroposterior motion is slightly decreased (anteroposterior ejection fraction 17%). In parallel with previous data, right ventricular function appears to be decreased with an apical predominance

In intracardiac shunts, the main driver of RV geometric and functional remodeling is the altered loading conditions of the ventricle, whereas in complex malformations of the heart, such as tetralogy of Fallot (ToF), the RV myoarchitecture is primarily affected. In contrast with healthy individuals, in ToF patients, a prominent middle layer with circumferentially oriented myofibers is also found, similar to the LV architecture. Consequently, we may hypothesize a relative dominance of radial RV function. In the current era of ToF management, total surgical repair is performed in the first years of life. Still, pulmonary regurgitation as a consequence of the repair procedure is a common finding. This volume overload of the RV may be tolerated for the long term; however, current data support the finding that the restoration of valve function must be performed to preserve RV function [27]. In the adult ToF population, global RV function is impaired, and decreased longitudinal function can be seen with apical predominance [24], along with compensatory increase in free wall radial shortening (Fig. 4) [28]. Clinical data suggests that the restoration of pulmonary valve function may have a beneficial effect on RV longitudinal function [29].

Arrhythmogenic cardiomyopathy (AC) is an inherited cardiomyopathy that predominantly affects the RV. While the genetic background of AC is established, other factors, such as the volume load of the RV, may precipitate the disease and accelerate progression [30]. Ventricular arrhythmias are the typical first clinical manifestation of the disease; however, RV functional impairment is also seen in more advanced stages [31]. Although in-depth analysis of RV morphology and mechanics reveals several markers of AC, diagnosis is still driven by clinical features, and we lack specific markers for subclinical disease. Whereas in overt stages, the dilation of the RV and globally reduced RV function are evident, in early-phase AC, normal RV volumes and slightly reduced RV global function are expected, with maintained longitudinal shortening [32]. The regional assessment of RV longitudinal function suggests that the basal (subtricuspid) strain may be a marker of subclinical involvement [33].

## Heart failure with preserved and reduced left ventricular ejection fraction

In heart failure with preserved ejection fraction (HFpEF), it has been shown that more than 50% of total deaths are attributable to right heart failure [34]. In the presence of LV diastolic dysfunction and the loss of left atrial compliance, a pulsatile overload burdens the pulmonary venous system. Typical comorbidities may even exaggerate pulmonary vasoconstriction, resulting in a disproportional increase in pulmonary pressures compared to the left side. A recent position paper from the European Society of Cardiology emphasizes the importance of transverse (radial) motion of the RV as a potential screening parameter for pulmonary hypertension in patients with HFpEF [35].

In heart failure with reduced ejection fraction (HFrEF), right heart failure develops because of gradual increases in afterload due to pulmonary hypertension and because of tricuspid regurgitation accompanied by volume overload. Due to the given pericardial space, the enlargement of one ventricle will affect its counterpart. Moreover, in the presence of overload conditions, misalignment of interventricular interactions and normal cardiac cycle events can lead to mechanical dyssynchrony. Geometrically, this change often means that in the early stages, the bulging of the septum will be more prominent, and the space for RV volume will drop. This effect may imply a heavier dependence on longitudinal shortening of the free wall, as seen in other conditions in which a heavily distorted RV geometry is present [36]. RV failure develops when the RV begins to dilate and loses its longitudinal function. There are numerous outcome studies showing the predictive value of baseline longitudinal RV function, most recently by assessing RV longitudinal strain [37].

Changes in RV mechanical pattern in conduction abnormalities and during RV pacing also attract scientific interest. Pacing from the RV results in a contraction pattern that is similar to a left bundle branch block [38]. This effect again disintegrates ventricular interdependence and hampers the LV contribution to RV function. Regaining interventricular synchrony and improving RV function may be an important factor in the established beneficial effects after upgrading to cardiac resynchronization therapy from RV pacing [39].

The assessment of RV function by echocardiography is also key in the selection of candidate patients for ventricular assist device implantation. The presence of RV dysfunction is unequivocally associated with worse outcomes. Carluccio et al. investigated and followed up 200 patients with HFrEF but normal TAPSE [40]. RV free wall strain provided incremental prognostic value compared to the conventional measurement. Moreover, 3D echocardiography-derived RV ejection fractions were found to be superior to clinical risk factors and conventional echocardiographic measurements in predicting adverse outcomes in a patient population with various cardiac diseases [41]. These studies point to the incremental value of advanced techniques, including both diagnostic and prognostic benefits, in heart failure patients. However, the incorporation of non-longitudinal motion directions and geometrical analysis based on 3D echocardiography remains to be tested.

## Cardiac surgery and heart transplantation

**Fig. 5:**
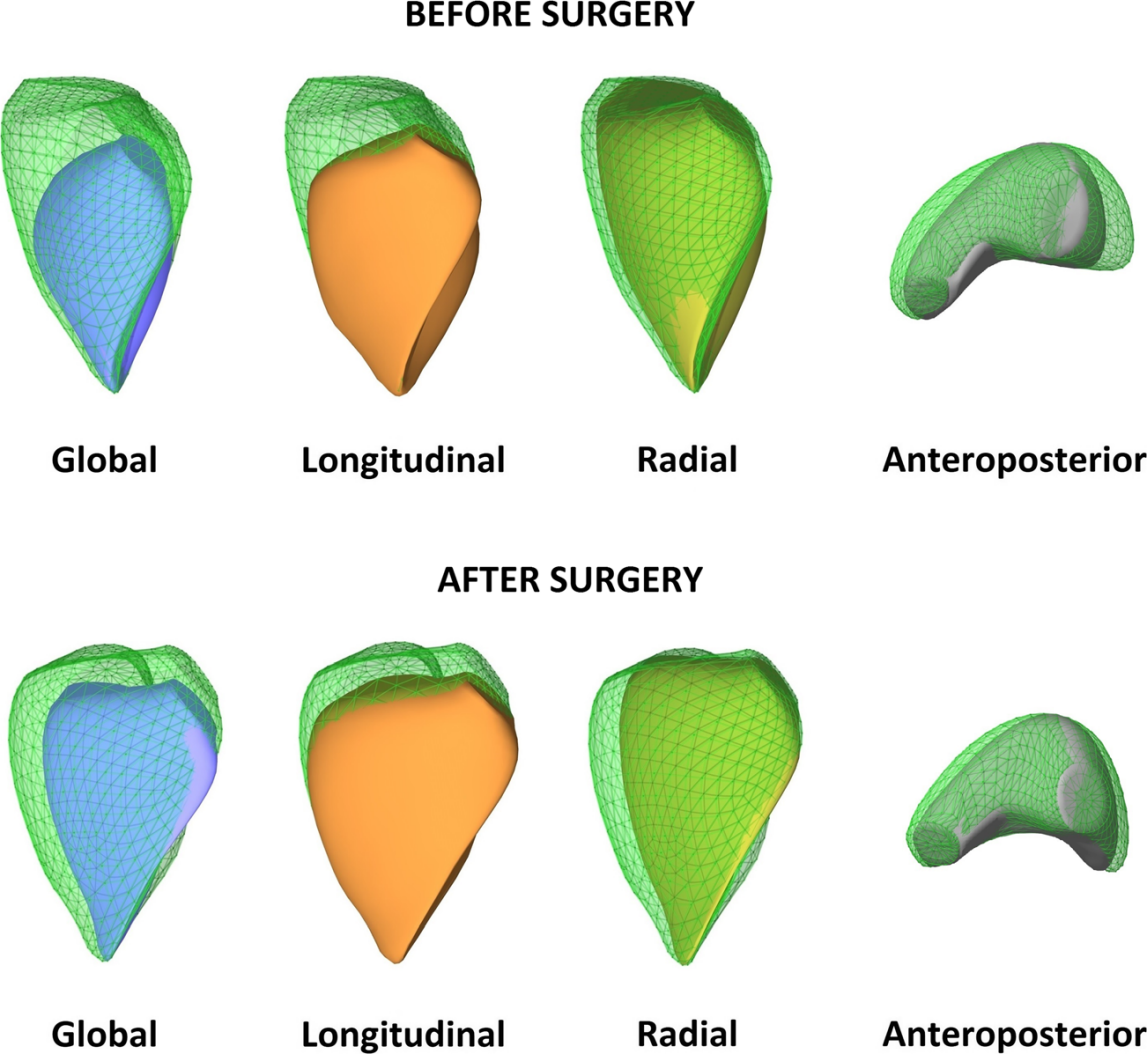
Patient with severe mitral regurgitation: before and following surgical mitral valve repair. Right ventricular dimensions and global function are in the normal range (end-diastolic volume 116 mL, ejection fraction 61%); however, the patient has an estimated peak pulmonary artery pressure of 47 mmHg, marking right ventricular pressure overload. Longitudinal function is preserved (longitudinal ejection fraction 29%) along with anteroposterior shortening (AEF 23%), while radial function is decreased (radial ejection fraction 13%). Following surgery, a functional shift can be observed in right ventricular function, while geometry is unaffected (right ventricular end-diastolic volume 107 mL): longitudinal and anteroposterior is markedly decreased (longitudinal ejection fraction 14%, anteroposterior ejection fraction 14%) while radial shortening increases (radial ejection fraction 28%), maintaining global function (ejection fraction 56%)

Left-sided valvular diseases subsequently affect the right heart through the varying burden of pressure- and/or volume overload. However, RV function is commonly found to be significantly altered following cardiac surgeries, such as coronary artery bypass grafting (CABG), surgical valve repair, or heart transplantation [6, 42]. The most prominent change is a decline in longitudinal shortening, even if the global RV function is preserved [43]. This decline in long-axis RV function is persistent and independent of the side of the surgical procedure. Majority of studies emphasizes the role of pericardiotomy in the reduction of RV long-axis movement because pericardial constraint and ventricular interdependence can be hampered by the opening of the pericardial sac. Notably, RV systolic function (ejection fraction and stroke volume) is mostly maintained regardless of the surgery, as radial RV contraction might compensate for the decline in longitudinal shortening (Fig. 5) [43, 44]. In addition to the significant effect of the pericardial incision, its extent also influences postoperative RV function along the long-axis; a less prominent decline was reported following minimally invasive surgical valve procedures than after full sternotomy [45, 46]. These findings suggest that minimally invasive approaches tend to have a less deleterious effect on longitudinal RV function and that preservation of the pericardium might be beneficial. Once the pericardium is disrupted, the RV contraction pattern will be permanently altered, even if pericardial repair is performed [47].

The evaluation of RV function following orthotropic heart transplantation deserves special attention because RV failure is still one of the major determinants of cardiac complications and mortality during the early postoperative period [48]. The alterations of the RV contraction pattern and the superiority of radial contractions compared to longitudinal shortening can be also observed in these patients [6]. However, the magnitude of longitudinal functional decline tends to be greater, and the recovery appears to be much slower than that is observed after other cardiac surgeries [42, 49]. Beyond the previously discussed factors, additional factor may be involved; hence, posttransplantation RV function is determined by a more complex interaction among the donor, the operation, and the recipient.

In conclusion, the decline in RV longitudinal function after cardiac surgery does not necessarily indicate a reduction in global RV systolic function. Therefore, reporting of RV parameters that also incorporate radial function is highly recommended [50].

## Athlete’s heart

Regular exercise training is associated with significant changes in cardiac dimensions and function, which are also referred to as athlete’s heart [51]. Traditionally, it was believed that the RV is only exposed to volume overload due to increased cardiac output; however, recent data also suggest the presence of significant pressure overload [52]. The overall effects of these hemodynamic changes are still a matter of debate, considering the growing evidence for exercise-induced RV injury and AC [30].

Although very few prospective follow-up studies were performed in the field, current data suggest that the volumetric adaptation of the RV to exercise is more pronounced than that of the LV and that the eccentric type of hypertrophy is expected at all levels of training [53]. Resting global RV function is often in the low-normal range and data suggests a relative decrease in radial shortening along with maintained or increased longitudinal function [7, 54]. Following ultraendurance sport events, however, the RV dimensions may increase, and decreased ejection fractions and GLS can be seen [54]. In the context of the concept of mixed hemodynamic RV overload in exercise, these results are consistent with the findings of pathological RV volume and pressure overload. As previously discussed, volume overload of the RV is associated with relatively increased longitudinal function [24], whereas RV pressure overload primarily affects radial shortening, with prominently decreased bellows motion [13]. When the exercise load of the RV passes a certain level and genetic predisposition is also present, RV damage may develop [30].

## Summary and future perspectives for RV research

In our review, we have provided a brief summary of the existing literature regarding the mechanical adaptation of the RV to different overload conditions. The RV, which is often referred to as the “forgotten chamber”, has emerged as an important factor and prognosticator for several cardiovascular conditions. The avoidance of RV failure is now a major and explicit goal, for instance, in pulmonary hypertension, HFpEF, and CHD patients.

Echocardiography is the first-line modality for assessing and monitoring RV morphology and function. However, 2D approaches fail to provide a good estimation of the non-longitudinal contraction of the chamber. Notably, the subepicardial layer of the RV myocardium consists of circumferentially oriented myofibers, which play a significant role in the complex contraction pattern. Nevertheless, further prospective studies are needed to assess the clinical value of in-depth characterization of RV mechanics. While pressure overload seems to affect the RV in a uniform way, the mechanical characterization of a volume-overloaded RV is more complex. Beyond contractility, the preload and afterload determine RV function, and the effect of pericardial constraint should not be neglected. Surgical procedures induce an instant shift in the functional pattern.

To provide a clinical recommendation on the assessment of RV function, it is important to emphasize that the sole evaluation of longitudinal shortening does not grant sufficient information concerning the majority of RV-related pathological conditions (Table 1). If applying 2D echocardiography, a comprehensive approach by multiple parameters is suggested, involving those measures that conventionally refer to the longitudinal shortening (TAPSE, tissue Doppler imaging) and, importantly, adding others that will at least partly incorporate radial shortening (FAC). The incremental diagnostic and prognostic value of speckle tracking-derived longitudinal strain is established for the assessment of the RV as well, and should be also implemented. However, 3D echocardiography-based geometrical and functional RV characterization may represent a real breakthrough in both clinical and research perspectives. Several software solutions by big vendors and by custom development enter the arena to also quantify the non-longitudinal component of the RV mechanical pattern.

**Table 1:** Comparison of the mechanical properties of right ventricular (RV) contraction in different physiological and pathological conditions and value of conventional and advanced echocardiographic techniques in the evaluation

	RV mechanical pattern	Value of assessing longitudinal shortening by 2D echocardiography	Added value of 3D echocardiography and related advanced techniques
Normal conditions	Comparable contribution of longitudinal and radial shortening	++	++
RV pressure overload	Early decrease in radial shortening (free wall basal segment predominance)	+	+++
RV volume overload	Increase in longitudinal shortening	++	++
CHD	Depending on the pathology	+(+)	+++
Arrhythmogenic cardiomyopathy	Mainly segmental differences	+	++
HFpEF	Early decrease in radial shortening	+	+++
HFrEF, pre-LVAD implantation	Depending on pressure- and/or volume overload	+(+)	++
Post-cardiac surgery, HTX	Increase in radial shortening, decrease in longitudinal shortening	+	+++
Athlete’s heart	Increase in longitudinal shortening, decrease in radial shortening	++	++

*2D* two-dimensional, *3D* three-dimensional, *CHD* congenital heart disease, *HFpEF* heart failure with preserved ejection fraction, *HFrEF* heart failure with reduced ejection fraction, *HTX* heart transplantation, *LVAD* left ventricular assist device

Using advanced imaging modalities, the knowledge gap in our understanding of the adaptation of RV shape and function appears to be narrowing. As for the LV, segmental and subsequent motion pattern analyses are the next steps in RV research activity.

## References

[1] Ghonim S, Voges I, Gatehouse PD, Keegan J, Gatzoulis MA, Kilner PJ, Babu-Narayan SV (2017). Myocardial architecture, mechanics, and fibrosis in congenital heart disease. Front Cardiovasc Med.

[2] Buckberg G, Hoffman JI (2014) Right ventricular architecture responsible for mechanical performance: unifying role of ventricular septum. J Thorac Cardiovasc Surg 148 (6):3166–3171 e3161–3164. 10.1016/j.jtcvs.2014.05.044, 3171.e4 10.1016/j.jtcvs.2014.05.044 24973008

[3] Geva T, Powell AJ, Crawford EC, Chung T, Colan SD (1998). Evaluation of regional differences in right ventricular systolic function by acoustic quantification echocardiography and cine magnetic resonance imaging. Circulation.

[4] Kukulski T, Hubbert L, Arnold M, Wranne B, Hatle L, Sutherland GR (2000). Normal regional right ventricular function and its change with age: a Doppler myocardial imaging study. J Am Soc Echocardiogr.

[5] Sakuma M, Ishigaki H, Komaki K, Oikawa Y, Katoh A, Nakagawa M, Hozawa H, Yamamoto Y, Takahashi T, Shirato K (2002). Right ventricular ejection function assessed by cineangiography—importance of bellows action. Circ J.

[6] Lakatos BK, Tokodi M, Assabiny A, Toser Z, Kosztin A, Doronina A, Racz K, Koritsanszky KB, Berzsenyi V, Nemeth E, Sax B, Kovacs A, Merkely B (2018). Dominance of free wall radial motion in global right ventricular function of heart transplant recipients. Clin Transpl.

[7] Lakatos BK, Kiss O, Tokodi M, Toser Z, Sydo N, Merkely G, Babity M, Szilagyi M, Komocsin Z, Bognar C, Kovacs A, Merkely B (2018). Exercise-induced shift in right ventricular contraction pattern: novel marker of athlete’s heart?. Am J Physiol Heart Circ Physiol.

[8] Matyas C, Kovacs A, Nemeth BT, Olah A, Braun S, Tokodi M, Barta BA, Benke K, Ruppert M, Lakatos BK, Merkely B, Radovits T (2018). Comparison of speckle-tracking echocardiography with invasive hemodynamics for the detection of characteristic cardiac dysfunction in type-1 and type-2 diabetic rat models. Cardiovasc Diabetol.

[9] Kalam K, Otahal P, Marwick TH (2014). Prognostic implications of global LV dysfunction: a systematic review and meta-analysis of global longitudinal strain and ejection fraction. Heart.

[10] Addetia K, Maffessanti F, Yamat M, Weinert L, Narang A, Freed BH, Mor-Avi V, Lang RM (2016). Three-dimensional echocardiography-based analysis of right ventricular shape in pulmonary arterial hypertension. Eur Heart J Cardiovasc Imaging.

[11] Lakatos B, Toser Z, Tokodi M, Doronina A, Kosztin A, Muraru D, Badano LP, Kovacs A, Merkely B (2017). Quantification of the relative contribution of the different right ventricular wall motion components to right ventricular ejection fraction: the ReVISION method. Cardiovasc Ultrasound.

[12] Tezuka F, Hort W, Lange PE, Nurnberg JH (1990). Muscle fiber orientation in the development and regression of right ventricular hypertrophy in pigs. Acta Pathol Jpn.

[13] Kind T, Mauritz GJ, Marcus JT, van de Veerdonk M, Westerhof N, Vonk-Noordegraaf A (2010). Right ventricular ejection fraction is better reflected by transverse rather than longitudinal wall motion in pulmonary hypertension. J Cardiovasc Magn Reson.

[14] Moceri P, Duchateau N, Baudouy D, Schouver ED, Leroy S, Squara F, Ferrari E, Sermesant M (2018). Three-dimensional right-ventricular regional deformation and survival in pulmonary hypertension. Eur Heart J Cardiovasc Imaging.

[15] Apitz C, Honjo O, Humpl T, Li J, Assad RS, Cho MY, Hong J, Friedberg MK, Redington AN (2012). Biventricular structural and functional responses to aortic constriction in a rabbit model of chronic right ventricular pressure overload. J Thorac Cardiovasc Surg.

[16] Kasimir MT, Seebacher G, Jaksch P, Winkler G, Schmid K, Marta GM, Simon P, Klepetko W (2004). Reverse cardiac remodelling in patients with primary pulmonary hypertension after isolated lung transplantation. Eur J Cardio-Thorac Surg : Off J Eur Assoc Cardio-Thorac Surg.

[17] Katz WE, Gasior TA, Quinlan JJ, Lazar JM, Firestone L, Griffith BP, Gorcsan J, 3rd (1996) Immediate effects of lung transplantation on right ventricular morphology and function in patients with variable degrees of pulmonary hypertension. J Am Coll Cardiol 27 (2):384–391 10.1016/0735-1097(95)00502-1 8557910

[18] Kuehne T, Saeed M, Gleason K, Turner D, Teitel D, Higgins CB, Moore P (2003). Effects of pulmonary insufficiency on biventricular function in the developing heart of growing swine. Circulation.

[19] Shah AS, Atkins BZ, Hata JA, Tai O, Kypson AP, Lilly RE, Koch WJ, Glower DD (2000). Early effects of right ventricular volume overload on ventricular performance and beta-adrenergic signaling. J Thorac Cardiovasc Surg.

[20] Rodes-Cabau J, Taramasso M, O'Gara PT (2016). Diagnosis and treatment of tricuspid valve disease: current and future perspectives. Lancet.

[21] Wald RM, Valente AM, Marelli A (2015). Heart failure in adult congenital heart disease: emerging concepts with a focus on tetralogy of Fallot. Trends Cardiovasc Med.

[22] Sanchez-Quintana D, Anderson RH, Ho SY (1996). Ventricular myoarchitecture in tetralogy of Fallot. Heart.

[23] Borgdorff MA, Bartelds B, Dickinson MG, Steendijk P, de Vroomen M, Berger RM (2013). Distinct loading conditions reveal various patterns of right ventricular adaptation. Am J Physiol Heart Circ Physiol.

[24] Dragulescu A, Grosse-Wortmann L, Redington A, Friedberg MK, Mertens L (2013). Differential effect of right ventricular dilatation on myocardial deformation in patients with atrial septal defects and patients after tetralogy of Fallot repair. Int J Cardiol.

[25] Brida M, Gatzoulis MA (2018). Pulmonary arterial hypertension in adult congenital heart disease. Heart..

[26] Moceri P, Bouvier P, Baudouy D, Dimopoulos K, Cerboni P, Wort SJ, Doyen D, Schouver ED, Gibelin P, Senior R, Gatzoulis MA, Ferrari E, Li W (2017). Cardiac remodelling amongst adults with various aetiologies of pulmonary arterial hypertension including Eisenmenger syndrome—implications on survival and the role of right ventricular transverse strain. Eur Heart J Cardiovasc Imaging.

[27] Geva T (2011). Repaired tetralogy of Fallot: the roles of cardiovascular magnetic resonance in evaluating pathophysiology and for pulmonary valve replacement decision support. J Cardiovasc Magn Reson.

[28] Stephensen S, Steding-Ehrenborg K, Munkhammar P, Heiberg E, Arheden H, Carlsson M (2014). The relationship between longitudinal, lateral, and septal contribution to stroke volume in patients with pulmonary regurgitation and healthy volunteers. Am J Physiol Heart Circ Physiol.

[29] Chowdhury SM, Hijazi ZM, Fahey JT, Rhodes JF, Kar S, Makkar R, Mullen M, Cao QL, Shirali GS (2015). Speckle-tracking echocardiographic measures of right ventricular function correlate with improvement in exercise function after percutaneous pulmonary valve implantation. J Am Soc Echocardiogr.

[30] James CA, Bhonsale A, Tichnell C, Murray B, Russell SD, Tandri H, Tedford RJ, Judge DP, Calkins H (2013). Exercise increases age-related penetrance and arrhythmic risk in arrhythmogenic right ventricular dysplasia/cardiomyopathy-associated desmosomal mutation carriers. J Am Coll Cardiol.

[31] Haugaa KH, Basso C, Badano LP, Bucciarelli-Ducci C, Cardim N, Gaemperli O, Galderisi M, Habib G, Knuuti J, Lancellotti P, McKenna W, Neglia D, Popescu BA, Edvardsen T, Eacvi Scientific Documents Committee EBm, external r, Eacvi Scientific Documents Committee EBm, external r (2017) Comprehensive multi-modality imaging approach in arrhythmogenic cardiomyopathy—an expert consensus document of the European Association of Cardiovascular Imaging. Eur Heart J Cardiovasc Imaging 18 (3):237–253. 10.1093/ehjci/jew229 10.1093/ehjci/jew229 PMC5837226 28069601

[32] Saberniak J, Leren IS, Haland TF, Beitnes JO, Hopp E, Borgquist R, Edvardsen T, Haugaa KH (2017). Comparison of patients with early-phase arrhythmogenic right ventricular cardiomyopathy and right ventricular outflow tract ventricular tachycardia. Eur Heart J Cardiovasc Imaging.

[33] Teske AJ, Cox MG, Te Riele AS, De Boeck BW, Doevendans PA, Hauer RN, Cramer MJ (2012). Early detection of regional functional abnormalities in asymptomatic ARVD/C gene carriers. J Am Soc Echocardiogr.

[34] Aschauer S, Zotter-Tufaro C, Duca F, Kammerlander A, Dalos D, Mascherbauer J, Bonderman D (2017). Modes of death in patients with heart failure and preserved ejection fraction. Int J Cardiol.

[35] Gorter TM, van Veldhuisen DJ, Bauersachs J, Borlaug BA, Celutkiene J, Coats AJS, Crespo-Leiro MG, Guazzi M, Harjola VP, Heymans S, Hill L, Lainscak M, Lam CSP, Lund LH, Lyon AR, Mebazaa A, Mueller C, Paulus WJ, Pieske B, Piepoli MF, Ruschitzka F, Rutten FH, Seferovic PM, Solomon SD, Shah SJ, Triposkiadis F, Wachter R, Tschope C, de Boer RA (2018). Right heart dysfunction and failure in heart failure with preserved ejection fraction: mechanisms and management. Position statement on behalf of the Heart Failure Association of the European Society of Cardiology. Eur J Heart Fail.

[36] Molnar AA, Kovacs A, Lakatos BK, Polos M, Merkely B (2018). Sinus of Valsalva aneurysm protruding intramurally into right ventricle: does size really matter?. Eur Heart J Cardiovasc Imaging.

[37] Nagy VK, Szeplaki G, Apor A, Kutyifa V, Kovacs A, Kosztin A, Becker D, Boros AM, Geller L, Merkely B (2015). Role of right ventricular global longitudinal strain in predicting early and long-term mortality in cardiac resynchronization therapy patients. PLoS One.

[38] Tanaka H, Hara H, Adelstein EC, Schwartzman D, Saba S, Gorcsan J (2010). Comparative mechanical activation mapping of RV pacing to LBBB by 2D and 3D speckle tracking and association with response to resynchronization therapy. JACC Cardiovasc Imaging.

[39] Kosztin A, Vamos M, Aradi D, Schwertner WR, Kovacs A, Nagy KV, Zima E, Geller L, Duray GZ, Kutyifa V, Merkely B (2018). De novo implantation vs. upgrade cardiac resynchronization therapy: a systematic review and meta-analysis. Heart Fail Rev.

[40] Carluccio E, Biagioli P, Alunni G, Murrone A, Zuchi C, Coiro S, Riccini C, Mengoni A, D'Antonio A, Ambrosio G (2018). Prognostic value of right ventricular dysfunction in heart failure with reduced ejection fraction: superiority of longitudinal strain over tricuspid annular plane systolic excursion. Circ Cardiovasc Imaging.

[41] Nagata Y, Wu VC, Kado Y, Otani K, Lin FC, Otsuji Y, Negishi K, Takeuchi M (2017) Prognostic value of right ventricular ejection fraction assessed by transthoracic 3D echocardiography. Circ Cardiovasc Imaging 10(2). 10.1161/CIRCIMAGING.116.005384 10.1161/CIRCIMAGING.116.005384 28174197

[42] Maffessanti F, Gripari P, Tamborini G, Muratori M, Fusini L, Alamanni F, Zanobini M, Fiorentini C, Caiani EG, Pepi M (2012). Evaluation of right ventricular systolic function after mitral valve repair: a two-dimensional Doppler, speckle-tracking, and three-dimensional echocardiographic study. J Am Soc Echocardiogr.

[43] Keyl C, Schneider J, Beyersdorf F, Ruile P, Siepe M, Pioch K, Schneider R, Jander N (2016). Right ventricular function after aortic valve replacement: a pilot study comparing surgical and transcatheter procedures using 3D echocardiography. Eur J Cardio-Thorac Surg : Off J Eur Assoc Cardio-Thorac Surg.

[44] Raina A, Vaidya A, Gertz ZM, Susan C, Forfia PR (2013). Marked changes in right ventricular contractile pattern after cardiothoracic surgery: implications for post-surgical assessment of right ventricular function. J Heart Lung Transplant.

[45] Dalén M, Oliveira Da Silva C, Sartipy U, Winter R, Franco-Cereceda A, Barimani J, Bäck M, Svenarud P (2018). Comparison of right ventricular function after ministernotomy and full sternotomy aortic valve replacement: a randomized study. Interact Cardiovasc Thorac Surg.

[46] Zanobini M, Saccocci M, Tamborini G, Veglia F, Di Minno A, Poggio P, Pepi M, Alamanni F, Loardi C (2017). Postoperative echocardiographic reduction of right ventricular function: is pericardial opening modality the main culprit?. Biomed Res Int.

[47] Lindqvist P, Holmgren A, Zhao Y, Henein MY (2012). Effect of pericardial repair after aortic valve replacement on septal and right ventricular function. Int J Cardiol.

[48] Yusen RD, Edwards LB, Dipchand AL, Goldfarb SB, Kucheryavaya AY, Levvey BJ, Lund LH, Meiser B, Rossano JW, Stehlik J, International Society for H, Lung T (2016). The Registry of the International Society for Heart and Lung Transplantation: thirty-third adult lung and heart-lung transplant report—2016; Focus theme: primary diagnostic indications for transplant. J Heart Lung Transplant.

[49] Kovacs A, Lakatos B, Nemeth E, Merkely B (2018). Response to Ivey-Miranda and Farrero-Torres “Is there dominance of free wall radial motion in global right ventricular function in heart transplant recipients or in all heart surgery patients?”. Clin Transpl.

[50] Badano LP, Miglioranza MH, Edvardsen T, Colafranceschi AS, Muraru D, Bacal F, Nieman K, Zoppellaro G, Marcondes Braga FG, Binder T, Habib G, Lancellotti P, Document r (2015). European Association of Cardiovascular Imaging/Cardiovascular Imaging Department of the Brazilian Society of Cardiology recommendations for the use of cardiac imaging to assess and follow patients after heart transplantation. Eur Heart J Cardiovasc Imaging.

[51] Olah A, Kovacs A, Lux A, Tokodi M, Braun S, Lakatos BK, Matyas C, Kellermayer D, Ruppert M, Sayour AA, Barta BA, Merkely B, Radovits T (2018). Characterization of the dynamic changes in left ventricular morphology and function induced by exercise training and detraining. Int J Cardiol.

[52] Naeije R, Vanderpool R, Dhakal BP, Saggar R, Saggar R, Vachiery JL, Lewis GD (2013). Exercise-induced pulmonary hypertension: physiological basis and methodological concerns. Am J Respir Crit Care Med.

[53] Arbab-Zadeh A, Perhonen M, Howden E, Peshock RM, Zhang R, Adams-Huet B, Haykowsky MJ, Levine BD (2014). Cardiac remodeling in response to 1 year of intensive endurance training. Circulation.

[54] La Gerche A, Burns AT, Mooney DJ, Inder WJ, Taylor AJ, Bogaert J, Macisaac AI, Heidbuchel H, Prior DL (2012). Exercise-induced right ventricular dysfunction and structural remodelling in endurance athletes. Eur Heart J.

